# The Immunology of SARS-CoV-2 Infection and Vaccines in Solid Organ Transplant Recipients

**DOI:** 10.3390/v13091879

**Published:** 2021-09-20

**Authors:** Dominika Dęborska-Materkowska, Dorota Kamińska

**Affiliations:** 1 Department of Transplantation Medicine, Nephrology and Internal Diseases, Medical University of Warsaw, Nowogrodzka 59, 02-006 Warsaw, Poland; dominika.deborska-materkowska@wum.edu.pl; 2 Department of Nephrology and Transplantation Medicine, Wroclaw Medical University, Borowska 213, 50-556 Wroclaw, Poland

**Keywords:** solid organ transplant recipients, COVID-19, cellular immunity, humoral immunity, vaccination

## Abstract

Since its outbreak in December 2019, the coronavirus disease 2019 (COVID-19) pandemic, caused by severe acute respiratory syndrome coronavirus 2 (SARS-CoV-2), led to an enormous rise in scientific response with an excess of COVID-19-related studies on the pathogenesis and potential therapeutic approaches. Solid organ transplant (SOT) recipients are a heterogeneous population with long-lasting immunosuppression as a joining element. Immunocompromised patients are a vulnerable population with a high risk of severe infections and an increased infection-related mortality rate. It was postulated that the hyperinflammatory state due to cytokine release syndrome during severe COVID-19 could be alleviated by immunosuppressive therapy in SOT patients. On the other hand, it was previously established that T cell-mediated immunity, which is significantly weakened in SOT recipients, is the main component of antiviral immune responses. In this paper, we present the current state of science on COVID-19 immunology in relation to solid organ transplantation with prospective therapeutic and vaccination strategies in this population.

## 1. Introduction

Since its outbreak in December 2019, the coronavirus disease 2019 (COVID-19), which is caused by severe acute respiratory syndrome coronavirus 2 (SARS-CoV-2) [1], has led to an enormous rise in worldwide morbidity and mortality. It was declared a pandemic by the World Health Organization (WHO) on 11 March 2020. The substantial spread of SARS-CoV-2 has led to dramatic consequences for medical systems and everyday life worldwide. The pandemic elicited a vast scientific response, with an extraordinary number of recent COVID-19-related studies of its pathogenesis and potential therapeutic approaches. 

Solid organ transplant (SOT) recipients are a heterogeneous population with long-lasting immunosuppression. Organ transplant recipients may be in relatively good clinical condition (similar to recipients of vascularized composite allografts, pre-emptive kidney allografts, or liver allografts after acute liver failure) or present severe long-lasting native organ failure with several comorbidities (similar to heart, lung, or most previously long-term-dialyzed kidney transplant recipients). 

The vast majority of SARS-CoV-2-infected patients are asymptomatic or have only mild symptoms. Approximately 5% progress to severe COVID-19 acute respiratory distress syndrome, septic shock, and multiorgan failure [2]. Immunocompromised patients are a particularly vulnerable population with a high risk of severe infection and high infection-related mortality rate [3]. SOT recipients were declared a group with a high risk for severe COVID-19 [4,5].

Immunosuppressant drugs may leave SOT recipients more susceptible to SARS-CoV-2 infection. However, it is unclear whether incidence rates differ for SOT recipients compared to the general population. A study on liver transplant recipients in Spain found transplant recipients had almost a two-times higher risk of developing COVID-19 compared with age- and sex-matched controls [6]. Data from a British registry showed that kidney transplant recipients (KTRs) were less often infected than patients on waiting lists, but mortality was 2.5-times higher (10.2% vs. 25.8%) [7]. The COVID-19-related death rate among KTRs was reported to be between 17.9% and 28% [8], with even a 60% short-term fatality rate in elderly KTRs [7,9]. Recipients older than 60 years of age, with concomitant cardiovascular disease or with deteriorated kidney allograft function, had a higher mortality risk [10]. It was hypothesized that in KTRs, impairments in kidney allograft function were a more significant mortality risk factor than immunosuppressive therapy. However, a recent multicenter study reported that the risk of COVID-19-related death was 78% higher in KTRs compared with hemodialysis patients after adequate adjustments for age, sex, frailty, and comorbidities. The mortality risk was radically higher during the first post-transplant year [11]. Results from the European Renal Association-European Dialysis and Transplant Association (ERA-EDTA) Registry from 2020 indicated that COVID-19-attributed mortality was 20.0% for patients on dialysis and 19.9% for KTRs [12].

During the first wave of the COVID-19 pandemic, the case fatality rate among hospitalized SOT recipients ranged from 13% to 29% in the initial reports [13,14,15], but recent data from a large, multicenter cohort study of critically ill adults with COVID-19 showed that the mortality rate of 40% in SOT recipients did not differ from 43% in the non-SOT group [16]. The authors postulated that the hyperinflammatory state that is caused by cytokine release syndrome during severe COVID-19 could be alleviated by immunosuppressive therapy in SOT patients. However, T cell-mediated immunity, which is significantly weakened in SOT recipients, is the main component of antiviral immune responses. Santeusanio reported that immunosuppression intensity and the degree of its reduction in a cohort of 38 KTRs following a COVID-19 diagnosis were not associated with mortality [8]. In a recent study of 482 SOT recipients who were hospitalized for COVID-19, the mortality rate was 20.5%, which was more related to age and underlying comorbidities than to immunosuppression intensity-related factors [17]. Another matched cohort study also showed that SOT recipients who were hospitalized with COVID-19 had similar outcomes as non-SOT patients [18].

This review presents the current state of science on COVID-19 immunology with regard to solid organ transplantation and prospective therapeutic and vaccination strategies in this population.

### Literature Search

The review of the literature about the immunology of SARS-CoV-2 infection and vaccines in solid organ transplant recipients was performed by both authors on PubMed with following keywords: “transplantation”, “transplant”, and “solid organ transplant” combined with at least one of the subsequent terms: “COVID-19”, “COVID”, “SARS-COV-2”, “COVID19” between 01JAN2020 and 25AUG2021. Only peer-reviewed, English-language research papers were included. The additional screen of the references included in original papers to obtain supplementary studies was performed. Due to an enormous number of published papers, only a subset of the papers were cited.

## 2. The Immunology of SARS-CoV-2 Infection

### 2.1. Mechanism of SARS-CoV-2 Infection

SARS-CoV-2 is a positive-sense single-stranded (ss) RNA virus. It spreads mainly through the respiratory system by binding to ACE2 receptors on the surface of human cells, such as lung surfactant-producing type 2 alveolar cells (type II pneumocytes) by the SARS-CoV-2 spike glycoprotein. After membrane fusion with host cells, SARS-CoV-2 releases its RNA into the cell cytoplasm, thereby allowing viral gene translation and replication [19]. SARS-CoV-2 is also able to infect various cells that express ACE2 receptors in many organs, leading to the involvement of the endothelium, kidney, heart, brain, liver, testes, eyes, and intestines [20,21]. Studies suggest that endothelium dysfunction during COVID-19 may exacerbate inflammatory and microvascular thrombotic processes [22]. 

### 2.2. Innate Immune Response to SARS-CoV-2

Host cells recognize specific viral molecular patterns (e.g., nucleic acids and proteins) via specific pattern recognition receptors. Endosomal viral RNA and DNA are recognized by Toll-like receptors (TLRs), and cytoplasmic viral nucleic acids are recognized by cytosolic melanoma differentiation-associated gene 5 (MDA5) receptors, retinoic-acid inducible gene I (RIG-I) receptors, and nucleotidyltransferase cyclic guanosine monophosphate-adenosine monophosphate (cGAS). This is followed by activation of the transcription factors nuclear factor κB (NF-κB) and interferon regulatory factor 3 (IRF3), leading to the secretion of IFN-α and IFN-β (IFN-I) and a wide range of proinflammatory cytokines, such as tumor necrosis factor α (TNF-α), interleukin-1 (IL-1), IL-6, and IL-18 [23]. These cytokines serve as main defense mechanisms against viral infection.

Under favorable conditions, rapid IFN-I production by infected cells occurs soon after infection, which limits viral replication within a few days [24]. Nevertheless, SARS-CoV-2 has developed several mechanisms to inhibit IFN-I secretion, mainly by translational evasion and shutdown of the IFN-I-dependent induction of interferon (IFN)-stimulated genes [25,26,27]. Significant impairments in IFN-I signatures were observed in patients with severe COVID-19 compared to mild or moderate cases, which was associated with persistent blood viral load and an exacerbated inflammatory response [28].

The innate immune response to SARS-CoV-2 is crucial for determining the fate of COVID-19 symptomatology. SARS-CoV-2 hampers the IFN pathway and stimulates the hyperproduction of proinflammatory cytokines and chemokines via NF-κB activation, which is conserved among COVID-19 variants [29,30]. Overall, an exuberant innate immunoinflammatory response is a hallmark of severe COVID-19.

### 2.3. Cytokine Storm in COVID-19

Cytokine storm, hyperinflammation, multiorgan failure, and acute respiratory distress syndrome were described in COVID-19 patients with high fever, dyspnea, lymphopenia, and high serum ferritin, D-dimers, C-reactive protein (CRP), and cytokines, including IL-1b, IL-6, and TNF-α [31,32]. Several studies showed that the severity of COVID-19 symptoms positively correlated with blood levels of proinflammatory cytokines and chemokines (e.g., IL-1b, TNF-α, monocyte chemoattractant protein 1 [MCP-1], MCP-3, cytosolic carboxypeptidase 2 [CCL2], CCL3, IL-2, sIL-2RA, IL-6, IL-7, IL-17, IL-18, granulocyte colony-stimulating factor [G-CSF], interferon gamma-induced protein 10 [IP10], macrophage colony-stimulating factor [M-CSF], and microprotein 1a [MIP-1a]) and anti-inflammatory cytokines (e.g., IL-10) [33,34,35,36,37,38]. A longitudinal study showed that levels of inflammatory cytokines remained elevated in severe COVID-19 cases but declined within days in patients with moderate disease [39]. The cytokine IL-6 was shown to be a valuable biomarker of COVID-19 severity and an indicator of viral load [40,41]. 

In a study of 494 KTRs, elevations of inflammatory markers (CRP, lactate dehydrogenase-LDH, and procalcitonin), cardiac injury markers (hs-troponin I), and thrombosis markers (D-dimers) were significantly associated with a higher risk of COVID-19-related mortality [42]. In a retrospective matched cohort of 30 SOT recipients with COVID-19, the highest CRP, D-dimer, procalcitonin, and LDH levels did not differ from the non-transplant group. SOT patients with critical disease had higher IL-6 levels compared with those with moderate and severe disease [43]. Early elevations of CRP, hs-troponin I, D-dimer, and IL-6 were significantly associated with severe disease and mortality in KTRs [44]. Lymphopenia and high levels of ferritin and IL-6 have been reported to be predictors of mortality [45,46]. No studies to date have provided more detailed innate immune profiling among SOT recipients.

### 2.4. Myeloid Cells

Driven by dendritic cells and macrophages, the dysregulated immune response to infection participates in the development of main COVID-19 syndromes. IL-1b, IL-6, and IFN-I/III from infected pulmonary epithelia contribute to macrophage activation and the further recruitment of monocytes, granulocytes, and lymphocytes from the circulation. Sustained IL-6 and TNF-α release by influent monocytes causes hyperinflammation cascades and cytokine release syndrome, a secondary hemophagocytic lymphohistiocytosis-like response followed by neutrophilic NETosis and microthrombosis, the induction of pathogenic Th1 and Th17 cell responses, and the recruitment of effector immune cells. Impairments in dendritic cell migration that leads to weakened antigen presenting cell function during SARS-CoV-2 infection may result in insufficient T cell activation and a lower number of virus-specific T cells in the lungs [47,48]. Deregulated NETosis was described as one of the mechanism of neutrophils’ response in viral infections and may lead to inadequately intense immune response with subsequent tissue damage. A severe COVID-19 inflammatory response contributes to a pro-NETosis state, so the potential therapies targeting NETosis (e.g., disulfiram, colchicine, dornase alfa, IL-1 receptor antagonists, anti-IL-6 etc.) potentially could decrease the risk of COVID-19 complications [49].

### 2.5. Natural Killer Cells

COVID-19 patients had a lower number of natural killer (NK) cells in peripheral blood, and high levels of IL-6 correlated with a lower number of NK cells [50]. Monocytes might impair NK cell recognition and thus kill SARS-CoV-2-infected cells. Natural killer cells are activated by immunoglobulin G1 (IgG1) and IgG3 antibodies during SARS-CoV-2 infection through Fc receptors. This suggests that antibodies that target IL-6 and TNF-signaling may restore NK cell functions in COVID-19 patients [48].

### 2.6. T Cells

T cells play an essential role in the antiviral response. CD8^+^ T cells kill infected cells, whereas CD4^+^ T cells activate B cells to produce antibodies. Lymphopenia with a significantly lower number of CD4^+^ T cells and a higher number of CD8^+^ T cells was described in severe COVID-19 cases, which correlated with mortality [50,51]. COVID-19-related impairments in CD4^+^ T cells promoted the excessive activation and possible subsequent exhaustion of CD8^+^ T cells [52]. The neutrophil-to-lymphocyte ratio (NLR) and neutrophil-to-CD8^+^ T cell ratio (N8R) were identified as powerful prognostic factors for COVID-19 patients [53].

A lower number of T cells at COVID-19 onset was reported to be a marker of progression to severe disease [54]. Lower levels of T helper cells, T suppressor cells, T regulatory cells, and CD16^+^CD56^+^ NK cells were reported in critical COVID-19 patients compared with non-critical COVID-19 patients, with impairments in the function of T and NK cells and very low levels of secreted cytokines [55]. However, Thieme reported that a robust anti-spike, membrane, and nucleocapsid SARS-CoV-2 protein T cell response was not associated with recovery in critical COVID-19 patients [56]. A spike-specific CD4^+^ memory T cell response was reported in >90% of convalescents over 4 months after infection. CoV-2-specific memory CD8^+^ T cells were detectable at ≥6 months in 50% of convalescents [57]. Two months after recovery from COVID-19, patients still presented lower levels of CD4^+^ T cells, B cells, and granulocytes. Previously hospitalized patients presented a prolonged proinflammatory response, with the lowest levels of CD8^+^ regulatory T cells, the highest levels of CD56^+^CD16^−^ NK T cells, and the promotion of a Th17-type phenotype [58]. 

Most studies of SOT recipients with COVID-19 also reported profound lymphopenia as in the general population [59]. The development of robust serological and functional T cell immune responses against SARS-CoV-2 was found among SOT recipients, similar to immunocompetent patients during early convalescence, with a trend toward lower SARS-CoV-2-reactive T cell frequencies [60].

A study of five KTRs with polymerase chain reaction (PCR)-confirmed COVID-19 found that they had reactive SARS-CoV-2-specific CD4^+^ and CD8^+^ T cells from 2 to 6 weeks after symptom onset and did not differ from patients on hemodialysis. All of them underwent a reduction of immunosuppression at the time of COVID-19 [61]. Eighteen KTRs with active COVID-19 infection had lower total lymphocytes and lower circulating memory CD4^+^ and CD8^+^ T cells compared with 36 matched KTRs without COVID-19. Fewer anergic and senescent CD8^+^ T cells were found in COVID-19 individuals, with no differences in exhausted CD8^+^ T cells or any CD4^+^ T cell subsets [62]. A detectable SARS-CoV-2-specific CD4^+^ T cell response was found in 57.1% and 47.4% of KTRs 4 and 6 months after COVID-19, respectively. The detectable SARS-CoV-2-specific CD8^+^ T cell response was 19.0% and 42.1% in these same cohorts [63]. In summary, the T cell-mediated anti-SARS-CoV-2 response generally did not differ from immunocompetent patients.

### 2.7. B Cells

SARS-CoV-2 provokes an immediate B cell response, with virus-specific antibody production during the first 3 weeks after symptom onset [64]. Neutralizing antibody titers are still detectable in the majority of recovered patients at least 8 months after SARS-CoV-2 infection [65]. The receptor binding domain (RBD) within the S protein is highly immunogenic, and anti-RBD antibodies block virus interactions with the entry receptor, ACE2 [66].

Memory B cells are able to undergo an immediate response to reinfection by generating new high-affinity plasma cells, which are essential for long-lasting immunity. Because of the short history of COVID-19, it is not yet possible to establish the extent of the long-term memory response. While neutralizing antibody titers decline modestly overtime, with an overall half-life of 90 days over the first 8 months after infection, anti-RBD binding antibody titers remain relatively stable over 6 months [57]. Moreover, the number of spike-specific memory B cells actually increased with time after symptom onset, whereas SARS-CoV-2-specific CD4^+^ T cells and CD8^+^ T cells declined, with a half-life of 3-5 months [57]. 

The adaptive immune response in SOT recipients generally resembles the response in immunocompetent patients, but antibody production and the T cell-mediated response may be inferior in SOT recipients. Immunosuppressive therapy as well as immune disturbances related to underlying diseases (e.g., chronic kidney disease) lead to impaired immune response to the SARS-CoV-2 infection. 

SOT recipients in general are able to mount anti-SARS-CoV-2 humoral response. Higher frequencies of activated B cells were reported in COVID-19 KTRs compared with non-infected recipients [62]. In a study of 161 SOT recipients, the anti-RBD antibody response 14 days after symptom onset was present in 90% of recipients and stable over time. The length of viral shedding was not influenced by antibody presence [67]. A matched analysis of 71 liver transplant recipients and immunocompetent controls after COVID-19 showed a lower incidence of anti-nucleocapsid IgG antibodies at 3 and 6 months [68]. Therefore, compared with immunocompetent patients, liver transplant recipients had a lower prevalence of anti-SARS-CoV-2 antibodies and a more pronounced decline of antibody levels. Moreover, T cell immune responses against SARS-CoV-2 was found among SOT recipients, early after infection [60], with a detectable SARS-CoV-2-specific T cell response in half of the recipients 4 and 6 months after COVID-19 [63]. SARS-CoV-2-reactive CD8+ T cells targeting membrane- and spike-protein were lower in SOT than in the general population. Nevertheless, polyfunctional CD8+ T cells as well as anti-nucleocapsid-protein-reactive CD8+ T cells did not differ significantly from non-immunocompromised individuals [69] Another study showed no differences between SOT and non-immunocompromised convalescents regarding the distinct SARS-CoV-2-reactive T cell response [60].

## 3. The Time of Viral Shedding among SOT Recipients with COVID-19

The peak of SARS-CoV-2 viral load in infected immunocompetent individuals occurs within the first week after symptom onset. Individuals experiencing an asymptomatic SARS-CoV-2 infection have a faster viral clearance compared to symptomatic individuals [70]. The time of virus shedding was reported to be longer among immunosuppressed kidney transplant recipients (over 28 days) and corresponded to a prolonged clinical course [71]. However, prolonged SARS-CoV-2 shedding was not universal among all immunocompromised SOT recipients. The time of viral shedding was not related to the total burden of immunosuppressive therapy. It was found that only older recipients or those with multiple comorbidities (diabetes, obesity, and rheumatologic disease) consistently presented delayed SARS-CoV-2 PCR clearance [72]. 

Prolonged COVID-19 in immunocompromised patients can increase the risk of the development of SARS-CoV-2 escape variants that can spread in the general population. In this context, SOT recipients with persistent viral shedding may generate more transmissible or more pathogenic SARS-CoV-2 variants and thus they should be prioritized for anti-COVID-19 vaccination [73].

## 4. Therapeutic Options in SOT Recipients in COVID-19

Many therapeutic options have been studied as potentially beneficial for the treatment of COVID-19. After initial reports of success, however, most of them failed to improve patient outcomes in randomized controlled trials. To date, there have been no randomized studies among SOT recipients [74]. Reports of specific medications that were used in SOT recipients are described below.

### 4.1. Antiviral Therapy in COVID-19

Remdesivir, a nucleotide analog that resembles adenosine triphosphate (ATP) and thus inhibits virus RNA synthesis, was considered a potential treatment option for RNA viruses, including SARS-Cov-2. Initial clinical reports indicated clinical and antiviral efficacy as a part of multidrug therapy in severe COVID-19 cases [75]. However, a recent meta-analysis showed that remdesivir had no effect on mortality in hospitalized COVID-19 patients [76] but may increase the recovery percentage and decrease the ventilation requirement rate. Based on data from clinical trials, the WHO advised against remdesivir use in COVID-19. Combinations of two other antiviral drugs, lopinavir and ritonavir, were found to exert no overall beneficial effect, and their use was strongly discouraged by WHO [77]. 

No drug–drug interactions were found between remdesivir and immunosuppressive drugs (e.g., calcineurin inhibitors—CNI, mycophenolic acid—MPA, glucocorticoids, and mammalian/mechanistic target of rapamycin (mTOR) inhibitors). 

Some small clinical trials of remdesivir use in SOT recipients were published. Fifty-seven moderate to severe COVID-19-positive KTRs received remdesivir as a part of COVID-19 management. A total of 14% cases died during the study, with 1.7% graft loss. The other recipients recovered, and no major adverse events were noted for remdesivir, including liver dysfunction [78]. A multicenter cohort study of 51 KTRs with COVID-19 who were treated with remdesivir found a mortality rate of 18.9%, with no significant hepatoxicity or systemic symptoms that resulted from the drug [79]. Forty-two KTRs with COVID-19 (19% with moderate disease and 43% with severe disease) received antiretrovirals (*n* = 10) or remdesivir (*n* = 8) as part of COVID-19 management. The mortality rate was 16.6%, and acute kidney injury was found in 24% of recipients at admission. The decrease in the estimated glomerular filtration rate was significantly more frequent in the remdesivir group (80%) compared with recipients with no antiviral treatment (29%). However, most patients exhibited the restoration of baseline kidney function within 1 month of discharge [80].

The successful management of severe COVID-19 pneumonia was reported in liver transplant recipients early after transplantation and treatment with remdesivir and convalescent plasma [81]. Another liver transplant recipient who suffered from COVID-19 with encephalopathy was successfully treated with remdesivir and convalescent plasma [82]. A case of a 67-year-old female with respiratory failure that was attributed to COVID-19 was described 1 year after cardiac transplantation. A reduction of immunosuppression with supportive treatment, including convalescent plasma, remdesivir, and dexamethasone, resulted in the resolution of her symptoms within days [83]. Additionally, two heart transplant recipients were successfully treated with a combination of dexamethasone and remdesivir [84]. 

In conclusion, remdesivir was reported to be safe in SOT recipients, but its use may result in at least the temporary worsening of kidney allograft function. 

### 4.2. Ivermectin

Ivermectin, an antiparasitic drug, can potentially act upon some viruses by altering ionic balance between the internal and external environments, resulting in osmotic lysis [85]. The neurotoxicity of ivermectin, especially at high doses, may limit its clinical use. Because of biased results from clinical studies, the WHO recommended against the use of ivermectin in COVID-19 patients except in clinical trials [77]. Ivermectin is not recommended in SOT recipients because it is known as a cytochrome P450 inducer that potentially alters CNI drug levels.

### 4.3. Immunotherapy to Reduce Cytokine Storm and Inflammatory-Mediated Organ Damage in COVID-19

Patients who succumb to severe COVID-19 often present an imbalanced immune response with exacerbated inflammation and dysregulated T cell activation and other counteracting activities [86]. Thus, the use of anti-cytokine and immunomodulatory medication was postulated to diminish inflammatory-mediated organ damage in the general population and SOT recipients. Immunomodulation has appeared as a promising option for SOT recipients with severe COVID-19 illness, but the available evidence is mainly restricted to the anti-IL-6 drug tocilizumab [87]. 

The potential benefits and hazards of immunomodulatory therapeutic options for COVID-19 in the general population relative to SOT recipients are discussed below.

#### 4.3.1. Glucocorticoids

Therapeutic doses of glucocorticoids affect both innate and adaptive immunity by affecting the production of proinflammatory cytokines (e.g., IL-1, IL-2, IL-6, IL-12, and IL-17), migration of macrophages/leukocytes into local inflamed sites, and regulation of Th1- and Th17-mediated cellular immunity and Th2-mediated humoral immunity. Glucocorticoids suppress cellular immunity but stimulate humoral immunity by altering the differentiation of CD4^+^ T cells and B cells and suppress IFN-I-mediated innate immunity by inhibiting their intracellular signaling pathways. Glucocorticoids increase the number of circulating neutrophils, enhance the opsonization of scavenger systems, and stimulate the phagocytosis of macrophages [88,89].

In COVID-19, glucocorticoids were reported to suppress virus-induced inflammation and subsequent organ damage. Dexamethasone at a dose of 6 mg/day for up to 10 days reduced mortality in COVID-19 patients who received either invasive mechanical ventilation or oxygen alone at randomization but not in patients who received no respiratory support [90].

SOT recipients are often treated chronically with glucocorticoids at least during the peri-transplant period. Thus, the use of dexamethasone or methylprednisolone as a part of anti-SARS-CoV-2 therapy is unquestionable. Most anti-COVID-19 therapies are based on an increase in temporary doses of glucocorticoids as a part of multi-drug treatment.

A case series of four heart transplant recipients showed that they were successfully treated with dexamethasone (with the addition of remdesivir in two patients) [84]. In a cohort of 32 lung transplant recipients with moderate/severe COVID-19, the mortality rate was 34%. Recipients received hydroxychloroquine (84%), azithromycin (75%), augmented steroids (44%), tocilizumab (19%), and remdesivir (9%) [91]. Two lung transplant recipients fully recovered after COVID-19-related acute respiratory distress syndrome after early treatment with high-dose corticosteroids [92].

#### 4.3.2. Chloroquine and Hydroxychloroquine

Hydroxychloroquine exerts immunomodulatory effects and is widely used for the treatment of rheumatologic diseases. Hydroxychloroquine augments antigen processing for MHC class I and II presentation, directly inhibits endosomal Toll-like receptor 7 (TLR7) and TLR9, enhances the activity of regulatory T cells, and has been used as an antiviral agent. After initial enthusiasm, however, hydroxychloroquine was reported to have no beneficial effect on any stage of COVID-19 [93].

Two patients who underwent liver transplant and were infected with SARS-Cov-2 in the early post-transplant period were reported to be successfully treated with hydroxychloroquine, methylprednisolone, tocilizumab, and convalescent plasma [94]. Twenty-seven lung transplant recipients with moderate/severe COVID-19 received hydroxychloroquine (84%) in the early period of the COVID-19 pandemic, but the treatment protocol changed over time according to the new evidence of COVID-19 therapy [91]. Based on current evidence, chloroquine and hydroxychloroquine use is contraindicated in COVID-19.

#### 4.3.3. Anti-IL-6 Receptor Antibodies 

IL-6 is a key regulator of cytotoxic T cell, monocyte, and B cell activity. The inhibition of IL-6 reduces innate and adaptive inflammatory responses [95,96]. 

Tocilizumab is a monoclonal antibody that targets the IL-6 receptor (both in soluble and membrane-bound forms) and thus blocks the signaling of IL-6. In COVID-19 patients, tocilizumab did not influence viral clearance or antibody production [97]. The Roche study of tocilizumab (COVACTA phase III clinical trial) did not meet its primary endpoint of improved clinical status in patients with COVID-19-associated pneumonia or the key secondary endpoint of lower patient mortality [98]. 

Sarilumab and siltuximab are two other human monoclonal antibodies against the IL-6 receptor. Phase III clinical trials showed no significant beneficial effects of sarilumab in COVID-19 patients [99]. One small study showed lower plasma CRP and IL-6 levels and lower mortality in COVID-19 patients who were treated with siltuximab. A small study showed that siltuximab reduced the risk of intensive care unit admission and mortality in patients with SARS-CoV-2 infection. A recently published Cochrane Database analysis of IL-6-blocking agents for treating COVID-19 showed that tocilizumab slightly reduced mortality but resulted in little or no clinical outcome improvement. Evidence of an effect of sarilumab is uncertain [100,101].

Two liver transplant recipients with COVID-19 in the early post-transplant period were successfully treated with tocilizumab (and hydroxychloroquine, methylprednisolone, convalescent plasma), with no signs of graft rejection [94]. Another case series of six SOT recipients reported a mortality rate of 33%, with no response to IL-6 blockade, remdesivir, and/or convalescent plasma [102].

#### 4.3.4. IL-1 Receptor Antagonists

Anakinra, a recombinant IL-1 receptor antagonist, was shown in small cohort studies to improve the survival rate of COVID-19 patients [103] and respiratory function [104]. Other clinical studies also showed that treatment with anakinra reduced plasma CRP levels and improved respiratory function in COVID-19 cases [105,106]. A recent meta-analysis of 15 anakinra trials in COVID-19 patients showed a beneficial effect on lowering mortality [107].

Some pediatric liver transplant recipients successfully received anakinra as a part of multi-drug therapy [108]. A study of KTRs showed no significant effect in terms of intensive care unit admission or respiratory secondary infections between anakinra- and tocilizumab-treated groups [109].

#### 4.3.5. Anti-TNF-α Antibodies

Infliximab, an anti-TNF-α monoclonal antibody, is widely used to treat autoimmune and inflammatory conditions. In severe COVID-19-induced cytokine storm syndrome with organ failure, infliximab was reported to exert a rapid and at least temporary decrease in proinflammatory cytokines and other inflammatory markers (e.g., CRP and LDH) with clinical improvement [110,111]. However, antibody responses to SARS-CoV-2 infection were shown to be attenuated in infliximab-treated patients with inflammatory bowel disease [112]. No studies of anti-TNF-α antibodies in the SOT population have been reported. 

#### 4.3.6. Janus Kinase Inhibitors

Janus kinase (JAK) signaling plays an essential role in the proinflammatory cytokine-mediated immune response during infection. Ruxolitinib is a selective inhibitor of JAK1 and JAK2, reducing the activity of multiple cytokines and chemokines. In recent study of 18 COVID-19 patients, ruxolitinib was successfully used to treat the hyperinflammatory state in 55% of the patients, regardless of prior steroid or tocilizumab therapy. However, a few patients exhibited severe evolution despite ruxolitinib therapy [113]. 

Baricitinib is a potent inhibitor of both JAK1 and JAK2 and also an inhibitor of numb-associated kinase (AAK1), which regulates the endocytosis of cells, thereby potentially inhibiting both SARS-CoV-2 entry and proinflammatory cytokine production. In COVID-19 patients, baricitinib reduced inflammatory markers, viral load, and mortality rate, with no serious adverse events [114]. A recent meta-analysis showed that JAK inhibitors play a potential role in reducing the risk of death in people with COVID-19 [115], but no studies of SOT recipients have yet been published.

### 4.4. Interferons as Therapeutics for COVID-19

Published data suggest that IFN-I deficiency in blood could be a hallmark of severe COVID-19 [28]. Animal models suggest that that the timing of IFN activation is protective in the early phase of infection but later becomes pathologic [116] via the upregulation of ACE2 in airway epithelia [117], among other mechanisms. Treatment with IFN-I in an animal model was successful only when applied earlier than 24 h after infection [116]. 

Treatment with the antiviral drug arbidol and IFNα-1b was reported to reduce the severity of illness in patients with moderate COVID-19 pneumonia [118]. A few small clinical trials in COVID-19 patients who received a subcutaneous injection of IFN-a2a or intranasal IFN-α2a or IFN-α2b reported faster recovery from the disease and faster virus clearance [119,120,121].

IFNγ was initially discovered as a potent antiviral agent [122]. The intranasal administration of IFNγ exerted a prophylactic effect in high-risk volunteers (i.e., medical workers and personnel in SARS-CoV-2 “red zones” in Russia) [123]. These authors also reported that the addition of IFNγ to multidrug antiviral therapy stabilized patients’ vital signs and resulted in no progression of pulmonary changes and no transfer to intensive care units [124].

No studies of SOT recipients have yet been published, but the use of IFN may not be advisable because of the potential risk of allograft rejection.

### 4.5. Convalescent Plasma

The efficacy of convalescent plasma therapy of COVID-19 is unclear. Although, most controlled trials have shown negative results, some studies and case reports of convalescent plasma therapy for COVID-19 have reported some effectiveness in COVID-19 patients with severe disease [125]. A recent meta-analysis showed that convalescent plasma as an adjunctive therapy could reduce the mortality rate among COVID-19 patients [126].

A case report of two liver transplant recipients reported that they were successfully treated for COVID-19 early after transplantation with convalescent plasma that was added to hydroxychloroquine, methylprednisolone, and tocilizumab [94]. Liver transplant recipients with severe pneumonia who received remdesivir and convalescent plasma experienced full recovery [82]. The successful treatment of three patients with convalescent donor plasma (with reduction or discontinuation of MPA in recipients) who developed severe COVID-19 directly after receiving kidney allografts was reported [127]. No acute rejection episodes after convalescent plasma therapy have been reported.

### 4.6. Monoclonal Neutralizing Anti-SARS-CoV-2 Spike Protein Antibodies

Monoclonal neutralizing antibodies bind SARS-CoV-2 spike protein and thus prevent viral attachment to ACE2 receptors. These antibodies were authorized for the treatment of high-risk patients with mild to moderate COVID-19 when administered in the early phase of infection [128]. The outcome of 16 SOT recipients (12 kidney transplant patients, one kidney-pancreas transplant patient, one kidney-liver transplant patient, and two heart transplant patients) who were given monoclonal antibodies (i.e., bamlanivimab, etesevimab, casirivimab, and imdevimab) was compared to an historical control SOT group. Neutralizing anti-SARS-CoV-2 monoclonal antibodies were shown to prevent acute respiratory failure in SOT patients with a good safety profile [129].

### 4.7. Adjustment of Immunosuppression

No randomized controlled studies have evaluated the management of chronic immunosuppression in SOT recipients with COVID-19. Based on experience with other infections, different therapeutic approaches have been proposed, with decreases in, or the discontinuation of, MPA and CNI doses and increases in corticosteroids [130]. Delayed SARS-CoV-2 PCR clearance was shown to be related to older age, multiple comorbidities, and solid organ transplant but not by overall immunosuppression burden [72]. Based on accumulating evidence, the adjustment of immunosuppression was less common during the second COVID-19 wave in Spain [131]. Various transplant society recommendations usually suggest stopping antiproliferative agents or continuing the use of standard immunosuppression [132].

Only a few episodes of acute rejections have been related to a decrease in immunosuppression, suggesting that in the case of COVID-19-related lymphopenia, the temporary cessation of CNI, MPA, or mTOR inhibitors may be safe even for 2 weeks [133]. In a cohort of 482 SOT recipients with COVID-19, acute rejection episodes occurred in only seven patients within 28 days [17]. There was one case of a kidney transplant recipient with moderate COVID-19 illness who was on belatacept-based immunosuppression and successfully received hydroxychloroquine, atazanavir, and a single dose of tocilizumab (with temporary discontinuation of MPA). Ten weeks after discharge, the patient received antithymocyte globulin, methylprednisolone, and rituximab because of biopsy-proven acute T cell- and antibody-mediated rejection. Four months later, the patient developed mild COVID-19 illness with a different lineage of SARS-CoV-2 [134].

Inflammatory damage can be weakened by anti-cytokine treatments. Some evidence suggests that maintenance immunosuppression agents can lessen COVID-19 symptoms. Some in vitro studies reported that the mTOR-related pathway plays an important role in the modulation of COVID-19 outcomes [135]. Based on hypothesized antiviral and immunomodulatory effects of cyclosporine, a study of KTRs showed that the discontinuation of MPA, together with a switch from tacrolimus to cyclosporine, reduced mortality with no graft function deterioration or signs of rejection [136]. One study of liver transplant recipients showed that tacrolimus dose maintenance was associated with better patient survival, which encouraged clinicians to keep the tacrolimus dose unchanged [137]. 

It was shown that mortality among SOT recipients was related to age and underlying comorbidities rather than immunosuppression intensity. However, in liver transplant recipients, immunosuppression that contained mycophenolate was an independent predictor of severe COVID-19, particularly at doses higher than 1000 mg/day. Such a severe COVID-19 outcome did not occur with mTOR inhibitors or CNI, and complete immunosuppression withdrawal showed no benefit [6]. A meta-analysis of data on 202 SOT recipients with COVID-19 suggested that maintaining immunosuppression might be safe in moderate and severe COVID-19. Moreover, tacrolimus could be specifically beneficial [138].

Recently, published recommendations for SOT recipients consist of a general approach based on disease severity:

- Mild disease (i.e., no symptoms or symptoms of upper or lower respiratory tract infection, with no hypoxia): supportive care only, no change in maintenance immunosuppression, and consider monoclonal neutralization.
- Moderate illness (i.e., hypoxia, requiring supplemental oxygen): dexamethasone (or equivalent steroid) up to 10 days and remdesivir for 5 days, and consider reducing or holding antimetabolite.
- Severe illness (i.e., mechanical ventilation or extra corporeal membrane oxygenation—ECMO requirement): dexamethasone (or equivalent steroid) up to 10 days, consider remdesivir for 5 days, and consider reducing or holding antimetabolite [74].

Multi-drug therapy during the COVID-19 pandemic creates a favorable situation for many drug–drug interactions to occur. QT monitoring is mandatory when hydroxychloroquine and azithromycin are combined. Azithromycin, lopinavir/ritonavir, remdesivir, favipiravir, chloroquine, hydroxychloroquine, and tocilizumab may interact with immunosuppressive drugs. Mycophenolate potentially interacts with lopinavir/ritonavir, with a need for dose reduction and close laboratory monitoring. Sirolimus may increase the level of atazanavir, and lopinavir/ritonavir and their combination are contraindicated. CNI increases blood levels of atazanavir, lopinavir/ritonavir, chloroquine, and hydroxychloroquine and may slightly decrease tocilizumab levels. Macrolides increase CNI levels, but azithromycin exerts minimal effects on the cytochrome p450 system [139]. Nevertheless, a case of acute kidney graft injury during COVID-19 treatment with azithromycin subsequent to tacrolimus increased levels of tacrolimus because of an azithromycin interaction with p450 enzyme [140].

## 5. Vaccination

In addition to public health measures, vaccination has emerged as a key tool for controlling the ongoing pandemic. A two-dose regimen of mRNA vaccine conferred 95% protection against COVID-19. Therefore, in December 2020, multiple regulatory agencies worldwide authorized the use of mRNA vaccines for SARS-CoV-2 [141]. After the start of the promotion of SARS-CoV-2 vaccines, the number of confirmed cases worldwide per week began to decline since the beginning of 2021. Unfortunately, previous reports have not addressed the prevention of SARS-Cov-2 infection in solid organ transplant recipients that have been excluded from major SARS-CoV-2 vaccine clinical trials [142,143].

Neutralizing antibodies are the part of adaptive immunity crucial for antiviral defense. Antibodies that target the viral receptor binding domain (RBD) in the S1 domain of the SARS-CoV-2 spike protein have been shown to exhibit a virus-neutralizing capacity [64,65,144]. After vaccination, the highest concentrations of serum and breast milk antibodies were observed with maximum about 29 days after the second dose [145]. 

Studies indicate immunocompromised people mount a reduced antibody response following a primary vaccine series, compared to immunocompetent vaccine recipients [146,147,148,149]. Immunogenicity to the SARS-CoV-2 vaccination can be assessed by quantifying antibodies to the spike receptor-binding domain and evaluating cellular responses. The preliminary results focused solely on antibody measurements following the first dose of vaccine and the timing of the antibody testing was variable, which could impact results [150]. The authors observed that less than 20% of patients had detectable antibodies, with the lowest response in those receiving antiproliferative medications and older individuals. Other studies indicate that kidney transplant recipients have an even weaker anti-SARS-CoV-2 antibody response, ultimately resulting in a low seroconversion rate 6.2 to 10.8% [151,152].

Given the absence of robust data to support the use of commercially available tests for measuring vaccine responses, it has been difficult to address their concerns. The data published to date do not give us the granular detail to develop recommendations from individual test results. Most commercially available serological assays are qualitative or, at best, semi-quantitative. Having a positive result may mislead a patient into thinking they are “safe” from infection when in fact titers are below protective titers. Conversely, a negative result may add to patient anxiety, despite contributions from untested factors, such as cellular immune responses. Furthermore, since the thresholds for protection and the impact of cellular responses are just now being established in healthy individuals, it further confounds our understanding of how to apply results to transplant recipients. 

Emerging data suggest that an additional COVID-19 vaccine dose in immunocompromised people enhances antibody response and increases the proportion who respond. In small studies, symptoms reported after the third dose were consistent with previous doses and the intensity of the symptoms was mostly mild or moderate. The prevalence of anti–SARS-CoV-2 antibodies was 0% before the first dose, 4% before the second dose, 40% before the third dose, and 68% 4 weeks after the third dose [153]. Among those who had no detectable antibody response to an initial mRNA vaccine series, 33–50% developed an antibody response to an additional dose. No serious adverse events were reported after administration of the third dose. No patients developed critical side effects requiring hospitalization [154]. 

There is a risk of the altered immunological response constituting a barrier in the effective vaccination against SARS-Cov-2 in the future and the patients may require additional or double doses of the vaccine as in the case of anti-hepatitis-B vaccination [154]. Additionally, if a lower response to SARS-CoV-2 vaccines is confirmed, public health authorities and transplant providers will need to have different thresholds for when these patients may safely return to more normal activity. 

These are not entirely surprising results, given that diminished antibody responses have been frequently described with other vaccines. Among kidney transplant recipients who received the influenza vaccine, only 33% of subjects reached seroprotective and seroconversion titers, with poorer responses in those less than 6 months post-transplant [155]. In another study, among a cohort of solid organ transplant recipients, a second dose does of influenza vaccine enhanced immunogenicity with a seroprotection rate after first dose of 69% versus 81% after the second dose [156]. Immune responses to viral infection in solid organ transplant recipients are not fully understood, but pioneer research indicates cell-mediated immunity is a critical factor for the control, prediction of viral replication, and recovery [157,158,159]. 

Novel immunodiagnostics to measure cellular immune response to SARS-CoV-2 are necessary to better evaluate the immune status of individuals and populations and assess emerging vaccines. Recent results indicate that SARS-CoV-2 Interferon-gamma release assay (IGRA) can be implemented in clinical laboratories to identify individuals with reactive T cells to SARS-CoV-2 [160]. In another study using IGRA, the authors observed that 78% of PCR-positive volunteers with undetectable antibodies showed T cell immunity against SARS-CoV-2 [161]. In the first study assessing humoral and T cell vaccine-induced responses to mRNA anti-SARSCoV2 vaccine in KTRs treated with belatacept, seroconversion occurred in very few patients, and T cell response in less than one-third of patients [162].

Some transplant professionals are advocating changes in patient management to improve vaccine responses, in particular the suspension of antiproliferative agents in anticipation of vaccination. However, unproven alterations in immunosuppression may ultimately be more detrimental than beneficial if changes increase the risk of rejection or provide no meaningful improvement in vaccine responses. One concern with vaccines in solid organ transplant recipients is the possible induction of alloreactivity and rejection. In one study of kidney transplant recipients, the authors showed that 12–17% of patients developed HLA alloantibody after an AS03-containing influenza vaccine. Although most of the reported anti-HLA antibodies had significantly declined or disappeared at 6 months, one patient suffered an acute antibody-mediated rejection and one developed a thrombotic microangiopathy [163]. In addition, a case-control study in heart transplant patients showed a significantly increased risk of rejection after the AS03 vaccine [164].

Then on the other hand, another study reported the development of de novo anti-HLA antibodies after receiving the influenza vaccine in kidney transplant recipients. Despite the fact that 60% of these antibodies were donor-specific, no episodes of antibody or cellular rejection were identified while these antibodies were detectable [155]. In addition, in a randomized trial in a wide range of transplant recipients, there was no correlation between the production of alloantibodies and influenza vaccine [165]. There is a knowledge gap regarding the immune mechanisms that confer protection against SARS-CoV-2 and the risk of acute rejection episodes in the post-vaccinated population of organ transplant recipients.

There are many indications that the adoption of the vaccine will change a lot in the peritransplant procedure and allow transplantation programs to be reopened. A few basic rules and timing of vaccination against COVID-19 in transplant recipients and candidates were proposed [132]. Due to quicker lowering of post-vaccination antibodies post-transplantation, it is anticipated that, in the case of the COVID-19 vaccine, an additional shot (or schedule) will be required. In August 2021, The Food and Drug Administration granted full approval to mRNA SARS-Cov-2 vaccine for people 16 and older. Following FDA’s decision Centers for Diseases Control and Prevention recommend an additional mRNA dose for moderately to severely immunocompromised people. This includes organ transplant recipients that are taking immunosuppressive therapy.

## 6. Conclusions

All available evidence indicates that SOT recipients with moderate or severe COVID-19 illness who are on chronic immunosuppressive therapy can mount SARS-CoV-2-reactive adaptive immune responses, both humoral and cellular. However, the precise immunologic characteristics of differences between immunocompromised SOT recipients and the immunocompetent population have not yet been characterized. The magnitude of the immune response to SARS-CoV-2 infection and vaccination, including kinetics and durability, should be examined. A detailed analysis of the “net state of immunosuppression” could help identify specific recipients who have a higher risk of SARS-CoV-2 infection. Such assessments may include the evaluation of not only immunosuppressive drug levels but also TTV and CMV replication, T- and B-lymphocyte subsets, soluble CD30, stimulated intracellular ATP levels, immunoglobulin, and complement levels [166]. 

Controlled randomized trials on anti-COVID-19 drugs as well as vaccines should also include SOT recipients to provide evidence of their efficacy in this group of patients.

## Data Availability

Not applicable.
